# Grounding Adaptive Cognitive Control in the Intrinsic, Functional Brain Organization: An HD-EEG Resting State Investigation

**DOI:** 10.3390/brainsci11111513

**Published:** 2021-11-15

**Authors:** Gian Marco Duma, Maria Grazia Di Bono, Giovanni Mento

**Affiliations:** 1 Institut de Neurosciences des Systèmes, Aix-Marseille Université, 13005 Marseille, France; 2 Department of General Psychology, University of Padova, 35129 Padova, Italy; mgdibono@gmail.com (M.G.D.B.); giovanni.mento@unipd.it (G.M.); 3 Padova Neuroscience Center (PNC), University of Padova, 35131 Padova, Italy

**Keywords:** cognitive control, functional connectivity, high-density EEG, machine learning, resting-state

## Abstract

In a recent study, we used the dynamic temporal prediction (DTP) task to demonstrate that the capability to implicitly adapt motor control as a function of task demand is grounded in at least three dissociable neurofunctional mechanisms: expectancy implementation, expectancy violation and response implementation, which are supported by as many distinct cortical networks. In this study, we further investigated if this ability can be predicted by the individual brain’s functional organization at rest. To this purpose, we recorded resting-state, high-density electroencephalography (HD-EEG) in healthy volunteers before performing the DTP task. This allowed us to obtain source-reconstructed cortical activity and compute whole-brain resting state functional connectivity at the source level. We then extracted phase locking values from the parceled cortex based on the Destrieux atlas to estimate individual functional connectivity at rest in the three task-related networks. Furthermore, we applied a machine-learning approach (i.e., support vector regression) and were able to predict both behavioral (response speed and accuracy adaptation) and neural (ERP modulation) task-dependent outcome. Finally, by exploiting graph theory nodal measures (i.e., degree, strength, local efficiency and clustering coefficient), we characterized the contribution of each node to the task-related neural and behavioral effects. These results show that the brain’s intrinsic functional organization can be potentially used as a predictor of the system capability to adjust motor control in a flexible and implicit way. Additionally, our findings support the theoretical framework in which cognitive control is conceived as an emergent property rooted in bottom-up associative learning processes.

## 1. Introduction

Imagine you have been driving the same road to reach your job for years. Likely, you have memorized the duration of each traffic light and can adjust your car’s speed automatically according to your implicit temporal expectation of the red light. Now, imagine that a new law decreases the duration of yellow lights from 4 to 3 s. Even if one does not consciously notice this difference, it will nevertheless not make it difficult to adapt one’s driving habits automatically according to this change. This is because our cognitive system is flexible enough to adapt to environmental changes without requiring a great deal of mental energy. Nevertheless, cognitive flexibility, a core function of cognitive control, has been traditionally considered as a top-down process able to guide actions based on both internal goals and the external context, therefore requiring volition and attention to down-regulate behavior [1,2,3,4,5,6,7]. However, a recent theoretical framework posits that cognitive control can be successfully driven by simple low-level associative learning processes, rather than necessarily requiring voluntarily controlled top-down processes [8,9,10,11,12].

In our recent work [13], we provided evidence supporting this hypothesis by showing that motor control can be shaped proactively by the implicit learning of stimulus temporal regularities. Specifically, we used the dynamic temporal prediction task (DTP) [14], a warned reaction-time (RT) paradigm in which the global presentation rate of the imperative stimulus is manipulated covertly in order to create “fast” (short-expectancy biased) or “slow” (long-expectancy biased) experimental blocks. We observed that both participants’ response speed and accuracy were shaped dynamically by the implicit learning of the global changes in the stimulus-presentation rate, so they were faster in the fast blocks and slower in the slow ones. Notably, because participants in the DTP are unaware of the implicit changes in the task speed, we argued that the cognitive flexibility underlying motor regulation is not exclusively enchained to voluntary control but can also be seen as an emergent property shaped by contextual factors. Furthermore, we showed that implicit flexibility is grounded in at least three functionally dissociable cognitive processes underlying adaptive motor control: expectancy implementation, expectancy violation and response implementation. These three separate computational stages were associated with a modulation of distinct event-related potential (ERP) components. Specifically, expectancy implementation was associated with a modulation of the pre-stimulus contingent negative variation (CNV) potential, while expectancy violation was related to a modulation of the omission detection (ODP) potential. Finally, the response implementation stage was associated with an increment of the post-stimulus P3 response. Importantly, all these effects were maximally expressed in the fast blocks, which induced participants to exert maximal implicit adjustment of behavioral performance (faster RTs). In our previous work [13], we had estimated the cortical generators of the above-mentioned ERP modulations, identifying three dissociable networks supporting expectancy implementation, expectancy violation and response implementation networks, respectively.

In the present study, we expanded upon our previous results by focusing on the question whether the intrinsic functional organization of the brain at rest may itself predict the capability to implicitly and flexibly adjust motor control as a function of contextual task demand. The possibility to foresee behavioral outcome or, even better, neural measures related to a given task by just decoding the intrinsic functional organization of the brain at rest has been revealed to be a promising methodological approach of the last decade. However, to the best of our knowledge, most of the studies have used functional magnetic resonance imaging (fMRI) [15,16] or were simply focusing on the scalp EEG activity [17,18]. Yet, the investigation of the EEG scalp activity does not allow us to make reliable spatial inferences about the underlying cortical generators, due to the volume conduction problem. In fact, in order to generate inferences related to brain areas or networks, the source reconstruction is necessary [19]. Moreover, the higher temporal and spectral resolution of the EEG compared to fMRI allows a more detailed investigation of the neural mechanism underlying cognitive processes. Here, we recorded high-density electroencephalography (HD-EEG, 128 channels) resting state activity and then computed source activations, from which we derived RS functional connectivity (RS-FC). We adopted a machine-learning approach (support vector regressions) to test the hypothesis of whether the RS oscillatory activity of the above-mentioned cortical networks, identified in our previous study [13], may predict both task-related behavioral performance and ERP modulations.

We observed that the intrinsic functional organization at rest in the response implementation and expectancy violation networks predicted the implicit adaptation of the motor response (task-related performance) in the theta and alpha band, respectively. Notably, we also observed that the RS-FC connectivity within a given network is able to predict its task-dependent activity modulation expressed in the related ERP modulation. Additionally, the topological properties (e.g., the degree, strength, clustering coefficient, and local efficiency) of each network of interest were studied using graph theory to explore the roles of single cortical nodes in predicting either behavioral or neural signatures of implicit motor control. We found converging evidence about the potential roles of the left motor area and the frontal right/parietal left regions as distinct nodes whose intrinsic functional organization may explain part of the variability in task performance. These results showed that the brain’s intrinsic functional organization can potentially be used as a predictor of the system’s capability to adjust motor control flexibly and implicitly. Additionally, our findings support the theoretical framework in which cognitive control is conceived of as an emergent property rooted in bottom-up associative learning processes, which does not necessarily need top-down voluntary processes to work efficiently. Finally, in the light of the investigated cognitive processes and the methodological approach, the present work adds two novelty points: (i) the study of the adaptive cognitive control using the DTP task, which is a task grounded on simple stimulus-response association; (ii) the prediction of task-related behavioral performance and networks modulation based on the resting state connectivity derived from the HD-EEG based source reconstruction, which provides an optimal compromise between temporal and spatial resolution to investigate at rest functional brain architecture.

## 2. Materials and Methods

### 2.1. Participants

Participants were the same included in our previous study [13]. The sample included 46 participants (mean age = 22.8 years, SD = 1.12, range 20–27, 8 males). For 40 of these 46 participants, we had both the resting state and the EEG data registered during the task. This implies that the prediction of task-dependent ERP modulations was possible only for this subset of 40 participants, whereas all participants’ data were used for behavioral performance prediction. All participants reported normal or corrected-to-normal vision and had no history of neurological and/or psychiatric disorders. All participants gave their informed consent before the experiment. All experimental procedures were approved by the Ethics Committee of the School of Psychology at the University of Padua (protocol n° 2536) and were conducted according to the principles expressed in the Declaration of Helsinki.

### 2.2. EEG Resting State Recording

For each participant, the RS HD-EEG activity was recorded before the task. We used a Geodesic high-density EEG System (EGI^®^ GES-300) with a pre-cabled 128-channels HydroCel Geodesic Sensor Net (HCGSN-128) and electrical reference to the vertex. EEG data were recorded during the entire experiment. The sampling rate was 500 Hz. The impedance was kept below 60 kΩ for each sensor. Continuous EEG resting state activity was recorded for two minutes while participants were passively watching the movie *Inscapes*, which has been specifically conceived and validated as a reliable tool to avoid drowsiness or sleepiness during task-free neuroimaging data collection [20].

### 2.3. Experimental Task

The experimental task was the same as described in [13]. Stimuli were presented on a 17-inch monitor at a resolution of 1280 × 1024 pixels. Participants were seated comfortably in a chair at a viewing distance of around 60 cm from the monitor. All participants performed a warned simple reaction time (RT) task adapted from an experimental paradigm previously employed from our lab to investigate voluntary and automatic temporal attention effects in adults and school-aged children [21]. This task, defined as Dynamic Temporal Prediction (DTP) [14,22] was originally conceived to investigate children’s behavioral performance in relation to either local or global probabilistic rules as two distinct sources of temporal predictability. We used a modified version adapted for ERP investigation here.

### 2.4. Trial Structure

**Figure 1:**
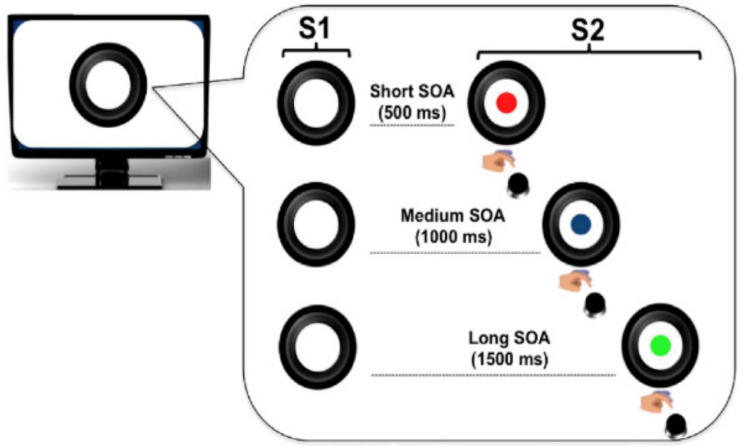
Trial structure. The circle (S1) warned participants on the presentation of the imperative S2 stimulus (a cartoon character; here represented with colored disks for illustrative purposes due to copyright restriction). Participants had to make speeded reaction times at S2 onset by pressing a button on the keyboard. The effect of local prediction was assessed by manipulating S1–S2 stimulus onset asynchrony (SOA) within each experimental block.

Each trial began with the display of a warning visual stimulus (S1), followed by the presentation of an imperative visual stimulus (S2) that stayed on the screen for a maximum of 3000 ms. S1 consisted of a picture of a black camera lens (see Figure 1) surrounded by a circle (total size of the stimulus: 840 × 840 pixels, 10.62° × 10.54° of visual angle). S2 consisted of a picture of a cartoon character, which was displayed centrally within the camera lens. The inter-trial-interval was randomly manipulated between 600 and 1500 ms. The task consisted of speeded target detection. Participants were required to press a button on the response box with the index finger of the dominant hand as quickly as possible on S2 occurrence.

#### 2.4.1. Local Predictive Context

To investigate the effect of the local predictive context on behavioral performance, the S1–S2 stimulus-onset-asynchrony (SOA) was varied trial by trial within each experimental block so that three possible fixed foreperiod (FP) intervals were created (Figure 1). These included a short (500 ms), a medium (1000 ms), or a long (1500 ms) FP, resulting in three discrete levels of hazard rate [23,24,25,26].

#### 2.4.2. Global Predictive Context

**Figure 2:**
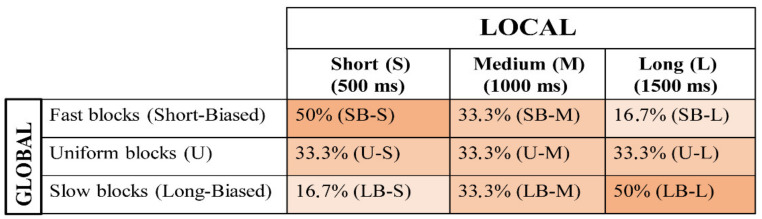
Experimental Design. The effect of global prediction was assessed by manipulating the between-block, a priori percentage of each SOA to create three probabilistic distributions in which the SOAs were equally distributed (uniform) or skewed toward the short (short-biased) or long (long-biased) SOA.

In order to assess the effect of the global changes on the predictive context, different probability distributions per each SOA interval were introduced and manipulated block-wise, as described below (see also Figure 2).

#### 2.4.3. Uniform (U) Blocks

In this block, a rectangular distribution of the three SOAs was used (33.3%, for each SOA) so that the probability of each SOA in the block was equally distributed. This type of distribution is the most classic probabilistic distribution employed in both adult [23,27,28] and developmental [21,29,30,31] SOA literature.

The use of an a priori uniform distribution has long been described to translate into a biased a posteriori temporal preparation. Indeed, as time goes by, the conditional probability of S2 onset increases exponentially in virtue of the fact that it has not occurred yet [23,32]. As a consequence, motor preparedness will be lowest at the shortest SOA and highest at the longest SOA.

#### 2.4.4. Fast Blocks (Short-Biased or SB)

In this case, an a priori distribution biased toward the short SOA was delivered. In particular, the relative percentage was 50%, 33.33%, and 16.67% for the short, medium, and long SOA, respectively. This distribution gives a faster stimulus frequency rate.

#### 2.4.5. Slow Blocks (Long-Biased or LB)

In this block, the relative percentage was 16.7%, 33.3%, and 50% for the short, medium, and long SOA, respectively. This kind of distribution, also known in the literature as aging distribution [32,33], is purposely intended to exacerbate the hazard-based increment of temporal expectancy as a function of SOA length. This distribution turned out to be a slowing down of the stimulus frequency rate.

#### 2.4.6. Experimental Design

The experimental manipulations yielded a factorial design in which either the SOA (short vs. medium vs. long) and the block type (fast vs. uniform vs. slow) factors were orthogonally contrasted to investigate the effect of local and global predictive context, respectively (Figure 2).

Each single block included 60 trials and was delivered three times, for a total of nine experimental blocks and 540 trials. Specifically, the total number of delivered trials were 90, 60 and 30, respectively, for short, medium and log SOA in the fast block; 60 trials for each SOA in the Uniform block and 30, 60 and 90 trials respectively for short, medium and log SOA in the slow block. All blocks were matched for sensorimotor requirements, as the visual stimuli and the required response were always the same across the experiment. The only differences were related to the changes in the predictive context experienced through the task. The total length of the experiment was about 25 min. It is important to note that participants were unbeknownst of both local and global manipulations since no explicit information was given about this. Furthermore, no pauses were introduced between blocks. Instead, a blank slide was inserted at the middle of each block to allow participants to rest. In this way we avoided participants to become aware about global changes occurring at any block switch. The block-type order was randomly sorted between subjects. This ensured that spurious effects due to introducing either local or global predictive contexts induced by a fixed SOA or block sequence did not bias the performance. To ensure that the experimental manipulation was effective in inducing implicit prediction, after completing the task we asked all participants if they realized that the task could change in speed, becoming faster or slower over time. None of the participants reported having noticed these changes, supporting that the change in the probabilistic context had an implicit impact on the behavior. Before starting the experimental session, participants were presented with a block of 20 training trials for each condition to ensure they understood task instructions. During training, all participants received feedback at every trial according to their performance. Specifically, a neutral yellow smile was displayed in cases in which either anticipatory (before target onset) or premature (<150 ms before target onset) responses were provided. A yellow smile was displayed if the RT was between 1000 and 1500 ms from target onset. Finally, a green smile was displayed if the RT was between 150 and 1000 ms. E-prime 2 software (Psychology Software Tools, Pittsburgh, PA, USA) was used to create and administer the stimuli. Behavioral data are available on Figshare public repository (https://doi.org/10.6084/m9.figshare.12248933, accessed on 14 November 2021).

### 2.5. Predicted Measures

**Figure 3:**
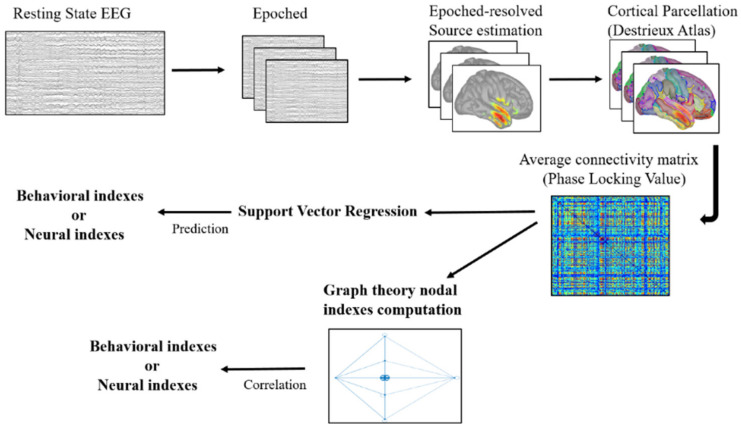
Graphical representation of the analysis pipeline. The present figure illustrates all the computational steps of the analysis pipeline of the present work starting from the resting state electroencephalography (EEG).

The behavioral and ERP data used in the present study were derived from a previously published database [13] and are publicly available (https://doi.org/10.6084/m9.figshare.12248933, accessed on 14 November 2021, https://doi.org/10.6084/m9.figshare.12246218, accessed on 14 November 2021). In the present study, we measured the mean accuracy and speed of response to the imperative stimulus for each participant. Only responses between 150 ms and 1500 ms from the target onset were considered correct responses and included in the RT analyses. To measure the implicit flexibility of motor control, we calculated the delta global index for each participant, which was the difference in his or her performance in the fast minus the slow blocks. We computed this measure for response speed (RT) and accuracy (non-premature responses). Moreover, we computed the inverse efficiency score (IES) to control for any speed–accuracy trade-off [14,34,35,36]. The IES is calculated by the following formula: IES = RT/(1 − PE), where RT is the subject’s average (correct) RT for the condition, and PE is the subject’s proportion of premature responses in the condition. In addition to behavioral data, we also targeted three distinct ERP modulations, which we found to be elicited by the DTP task in our original study [13]. Specifically, we identified the pre-stimulus modulation of the contingent negative variation (CNV), the omission detection potential (ODP) [13,31] elicited by the missed occurrence of an expected stimulus and the post-stimulus P3 response as neural signatures of expectancy implementation, expectancy violation and response implementation, respectively. Then, we performed machine-learning analyses to investigate whether RS-FC predicts both behavioral (RT, ACC and IES) and neural signatures (CNV, ODP and P3 modulation), separately. Moreover, we correlated these three behavioral and neural indexes with nodal graph-theory measures. All of the details are described in the following sections. The whole analysis pipeline is illustrated in Figure 3.

### 2.6. EEG Resting State Pre-Processing

Signal preprocessing of the resting EEG was performed through EEGLAB 14.1.2b [36]. The continuous EEG signal was first down sampled at 250 Hz and then bandpass-filtered (0.1 to 45 Hz) using a Hamming windowed sinc finite impulse response filter (cut-off frequency = −6 dB; roll-off = (0.05–45.05) Hz. The signal was successively segmented in epochs of 2 s. Extremely noisy (>350 μV) and flat (<1 μV) channels were automatically detected through the Trimoutlier EEGLAB plug-in. Epoched data were subjected to an automated bad-channel and artifact detection algorithm by using the TBT plugin implemented in EEGLAB. This algorithm identified the channels that exceeded a differential average amplitude of 250 μV and marked those channels for rejection. Channels that were marked as bad on more than 30% of all epochs were excluded. Epochs with more than 10 bad channels were also excluded. Data cleaning was performed by means of an independent component analysis (ICA) [37], using the Infomax algorithm [38] implemented in EEGLAB, obtaining 40 components. The resulting independent components were visually inspected in topography and time-series, and those related to eye blinks, eye movements, muscle and cardiac artifacts were discarded. The remaining components were then projected back to the electrode space to obtain cleaner EEG epochs. Finally, bad channels were reconstructed with the spherical spline interpolation method and the data were then re-referenced to the average of all electrodes [39,40].

### 2.7. Cortical Source Modelling

Epochs were imported in Brainstorm [41] to model the underlying cortical generators. We used the ICBM152 anatomical template to approximate the individual anatomy of each participant [42]. Co-registration of EEG electrodes position was performed via Brainstorm, by projecting the digitized EEG sensor positions GSN Hydrocel 128 E1 available in Brainstorm on the head surface. We then derived an EEG forward model using the three-layer boundary element method (BEM) from OpenMEEG implemented as a Brainstorm routine [43,44]. The source space was constrained to the cortex and modeled as a grid of 15,002 orthogonal current dipole triplets. We used sLORETA (default parameter setting) as a source model, one kernel for each subject as suggested in the resting state analysis pipeline with Brainstorm [45].

### 2.8. Network Definition and Functional Connectivity

**Figure 4:**
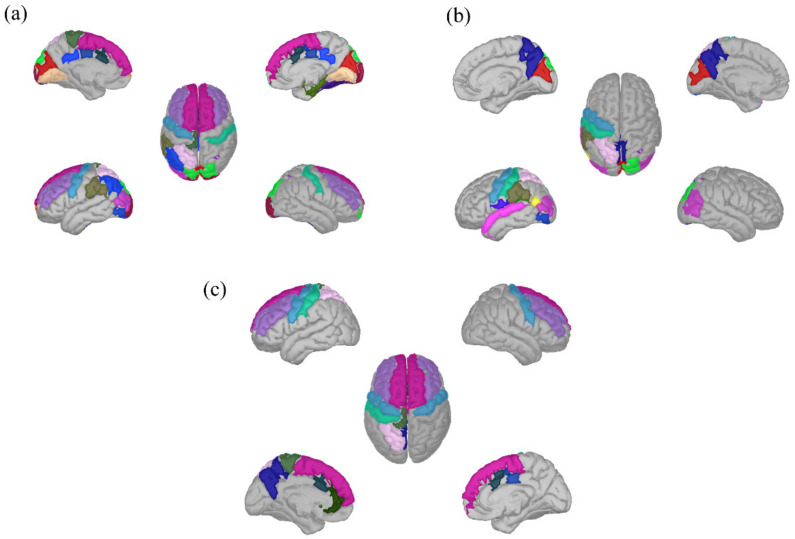
Networks of interest. The present figure represents the nodes included in each of the three networks of interest, namely expectancy implementation network (**a**), expectancy violation network (**b**) and response implementation network (**c**).

To compute resting state functional connectivity (RS-FC), we first parceled the cortical surface into 148 regions based on the Destrieux atlas [46], already implemented in the default anatomy of Brainstorm. Phase-locking value (PLV) [47] was computed in Brainstorm as a functional connectivity metric [48,49]. The phases of the two signals are extracted by the Hilbert transform and signed as ϕa and ϕb. The formula takes the average of phase angle differences between the two signals over time. In detail, we computed for each resting epoch the PLV value between all the atlas nodes, and then averaging across epochs, finally obtaining a N × N (N = number of regions in the Destrieux atlas) adjacency matrix for each canonical frequency bands (Delta (2–4 Hz), Theta (4–7 Hz), Alpha (8–12 Hz), Beta (13–30 Hz), Gamma (30–45 Hz)). The ROIs used to generate our target RS networks were derived from our previous work [13]. Specifically, in our previous study we reconstructed the cortical maps underlying the CNV, ODP and P3 effects as neural signatures of expectancy-implementation, expectancy-violation and response-implementation ERP effects, respectively (see Figure 4 for a graphic representation of the three networks). Here we used the brain areas identified by the source reconstruction of the three ERP effects as the node to construct our RS networks. This allowed us to spot three discrete networks which were named based on their relation to the computational process, namely, expectancy implementation, expectancy violation and response implementation networks. It is worth noting that we named our target network in this way to emphasize a direct link to cognitive processes elicited in performance of the present task identified in our previous study [13]. We are not claiming that for example, the expectancy implementation network can be completely generalized to every task involving expectancy. Even though we already discussed in our previous work the consistency between our results and the present literature related to cognitive flexibility and temporal expectancy, the reader should be aware that our networks are referred to cognitive processes elicited by our task. The nodes forming each circuit are fully listed in the Supplementary Table S1.

### 2.9. Support Vector Regression

In order to predict both behavioral performance and neural modulations (i.e., ERP activity) at the single-subject level, we used a machine learning approach by applying a support vector regression (SVR) model to the obtained resting state connectivity patterns. The SVR, defined by [50], was derived from the support vector machine (SVM) that was first introduced by [51] as a supervised learning model for classification problems. Thus, SVR can be considered a generalization of the SVM model for regression problems. For each of the three networks of interest and frequency bands, we created a dataset of connectivity patterns, one per subject. In this case, we have only one sample (i.e., the PLV value) per subject for the entire RS activity, thus our regression problem was implemented at the population level. In more detail, each subject-connectivity pattern was obtained by extracting the inferior triangular part of the PLV adjacency matrix. The target parameters to be predicted from the population-connectivity data were the following: task performance (delta-global RT/Accuracy/IES) at the behavioral level and ERP effects (CNV, ODP, P3) at the neural level. A leave-one-subject-out cross-validation (i.e., a leave-one-out cross validation scheme, implemented across population) was used to estimate the test generalization prediction accuracy. Specifically, training and test were performed a number of times (runs) equal to the population size (e.g., 46 subjects). For each run, we randomly selected and used data from all the subjects but one (e.g., 45) for training and the data from the discarded subject as a test sample. As suggested by [52], leave-one-out is preferred with dataset ≤ to 100 samples. By contrast, with colossal dataset it could increase the overfitting probability, and therefore other cross-validation approaches, as k-folds, can be used [52,53]. For these reasons we selected this cross-validation approach amongst other possible methods given our sample size dimension. Each target parameter that had to be predicted by the model was standardized (z-scored) in order to have zero mean and standard deviation one. Moreover, the connectivity data matrix for training was also standardized across subjects. We used the Matlab functions fitrsvm and predict, respectively, to train a linear SVR model (used with default parameters) and predict the target parameter of the test subject starting with its connectivity pattern. Matlab function cvpartition was used, at each run, for implementing the “leave one subject out” cross-validation scheme. The prediction accuracy was computed at the end of the cross-validation loop on the predicted parameter values for the test subjects. Specifically, we computed the Bayesian correlation between the vector containing the concatenation of the target value to be predicted at each run (i.e., one for each test subject) and the vector containing the concatenation of the corresponding predicted values. Thus, the prediction accuracy was expressed in terms of a correlation coefficient (see [54], for a similar procedure applied to fMRI data). We used Bayesian correlation since it provides a measure of the likelihood of the alternative hypothesis. Only positive correlations that were significant were reported as an index of a good-quality fitting. Note that negative correlations are an index of a very bad fitting and were just not considered. For the sake of completeness, the full results are given in an additional file on Open Science Framework (https://osf.io/xw8nq/, accessed on 14 November 2021). The analysis has been performed using the software JASP (https://jasp-stats.org/, accessed on 14 November 2021), reporting the Bayesian Factor (BF) in favor of the alternative Hypothesis H1 (positively correlated), according to which there is a correlation between the considered variables. Note that the reported BF values correspond to the assumption of a beta prior width = 0.5, but contextually, a BF robustness check will be reported in the following pictures in order to estimate the strength of the results. Considering the scale of interpretation of the Bayes factor (BF) given [55], here we only reported the BFs above √10, namely 3.162.

### 2.10. Graph Theoretical Analysis

In order to deeply investigate the results obtained from the first SVR analysis, we applied graph theoretical analysis to a new network of interest, defined as the ‘Intersection network’. This included all the ROIs within the response-implementation network that also belonged to the expectancy-implementation or the expectancy-violation networks. We pulled together all the ROIs that maximally covered the three considered networks (see Supplementary Materials, Table S1), starting from the ROIs within the Response Implementation network, from which it was possible, as SVR showed, to predict all the behavioral effects. Our aim was to study whether network topology properties expressed in terms of nodal indexes like degree, strength, clustering coefficient, and local efficiency, should be predictive of the performance during the task, at both the behavioral and neural level.

#### 2.10.1. Graph Construction

We used a single-subject-connectivity-matrix approach, as suggested by [56]. Thus, for each network of interest, frequency band, and subject, we constructed a graph and extracted graph measures (i.e., one global measure for graph construction and 4 local measures) by using the Brain Connectivity Toolbox (BCT) [57]. Graphs were constructed starting from the N × N adjacency matrix, where N is the number of ROIs included into the network under examination, using the proportional thresholding method. Note that graph connections were not binarized, in order to avoid a loss of information. More in detail, starting from a weighted adjacency matrix, we ranked the connections (i.e., the PLV values) in a descendent order and selected a specific percentage of the top ranked connections, expressed by a specific threshold. The selection of the graph threshold was performed by adopting a procedure based on controlling an important global measure (i.e., the small-word parameter) as a function of the selected threshold [56,58]. We computed the small-word parameter using the formulation in the [59], implemented in the BCT. Specifically, for each frequency band, we computed the small-word parameter on all the subjects and selected the larger threshold that preserved the small world network topology (i.e., mean small-world parameter > 1). This procedure discards the more defragmented graphs at the lower thresholds, which allow us to avoid information loss. Furthermore, it is a conservative procedure that maintains a connected graph and allows us to discard spurious connections [56,58].

#### 2.10.2. Graph Measures

All the measures were computed using the normalized weighted graphs obtained after thresholding. Specifically, after thresholding, we used the function weight conversion (used with the parameter option normalize), contained in the BCT [57] for normalizing the graph connectivity. This function scales all weight connections to the range (0, 1) by dividing the connection values to the maximal weight and should be done prior to computing some network parameters. Indeed, since network measures strictly depend on the mean of the weighted connections, weighted graphs obtained after thresholding need to be normalized in order to perform statistical analysis on the extracted measures.

The global measure (i.e., the small-world parameter) was computed for the graph construction, as above mentioned. Small-worldness (i.e., small-world parameter > 1) is a property characterizing graphs with dense local clustering and relatively few long-range connections, and it can globally account for both specialized (segregated) and distributed (integrated) information processing. Smallworld organized networks are characterized by a clustering coefficient C that is higher than the C of a randomly organized network (Crandom), and a short characteristic path length L that is similar to that of an equivalent random network (Lrandom) [60]. Formally, small-world networks show a ratio c defined as Creal/Crandom that has to be >1 and a ratio k defined as Lreal/Lrandom that has to be about 1 [60]. For each subject, the small-world indices were derived from the comparison of the real (measured) network with a random network including the same number of nodes, edges, and preserving the same degree distribution of the real network. Random networks were constructed by using the function randmio_und contained in the BCT [58].

In order to better distinguish between segregation and integration properties within brain networks, we extracted multiple local measures (i.e., nodal measures, one value per subject for each node within the graph) from each considered brain network modeled as a graph: (i) degree (or valency) of a graph node is the number of connections incident to the node, survived after thresholding [57,61], and could be interpreted as an index of integration; (ii) strength, which is computed for each node of the graph as the sum of the weights on its connections, and could be interpreted as an index of integration and synchronization of brain activity [57,61] (iii) local efficiency that provides an indication of how effectively information is integrated between the immediate neighbors of a given graph node; indeed, the topological organization of a network is directly related to its local and global efficiency, which jointly determine the network’s capability of integrating information effectively [57,61,62]; (iv) clustering coefficient, which is locally computed as the fraction of triangles around each individual node; it reflects the prevalence of clustered connectivity around individual nodes, and roughly corresponds to an index of segregation and specialization [57,61].

### 2.11. Correlation Analysis

We investigated whether there was a correlation between local graph indexes, extracted from the graphs of interest and the corresponding behavioral (i.e., delta global ACC/RT/IES) and ERP effects (i.e., CNV, ODP and P3). As mentioned above, this second step of analysis aimed at a deeper investigation of those functional connectivity networks from which it was possible to predict both behavior and neural effects. Indeed, this analysis was applied only in the Intersection network, which was previously defined. The study of the topological properties of the networks of interest, is an appealing opportunity to interpret the connectivity information driving SVR prediction in terms of segregation/integration processes (see [63], for a similar argument in fMRI data analysis). In particular, we performed Bayesian correlation analysis using the software JASP (https://jasp-stats.org/, accessed on 14 November 2021), reporting the Bayesian Factor (BF) in favor of the alternative Hypothesis H1, according to which there is a correlation between the considered variables. Note that the reported BF values correspond to the assumption of a beta prior width = 0.5, but contextually, a BF robustness check was also reported.

## 3. Results

In the following sub-sections, we reported all of the SVR results in predicting both the neural and behavioral indexes, starting from the PLV connectivity matrices of the networks of interest (i.e., the expectancy implementation, expectancy violation and response implementation networks) in each frequency band. Finally, we reported the Bayesian correlations between graph theory indexes and behavioral or neural indexes.

### 3.1. Predicting Behavioral Effects from the Networks of Interest

**Figure 5:**
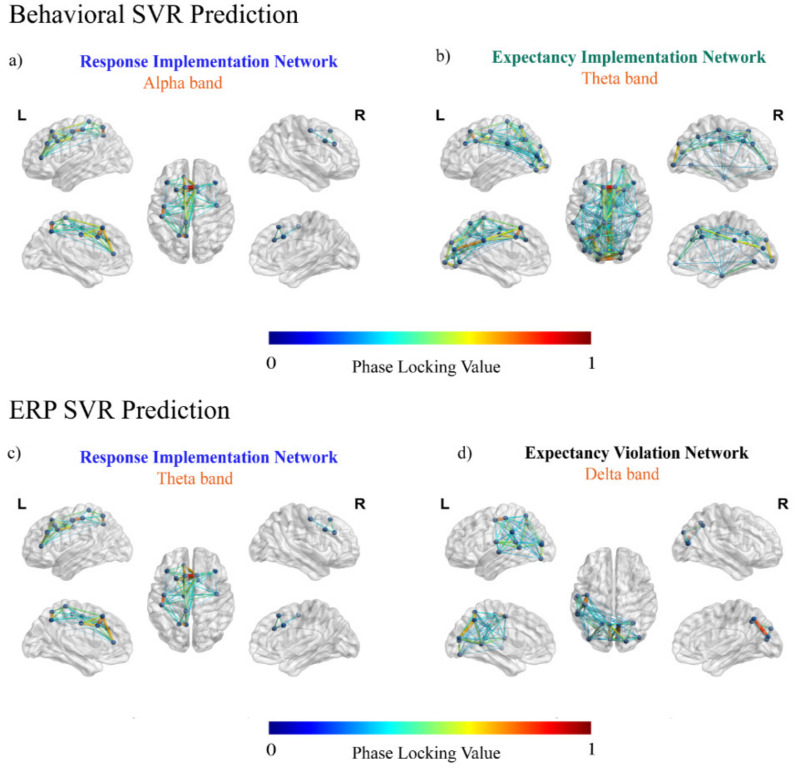
Resting state functional connectivity (RS-FC) predicting task-dependent behavioral and event related potential (ERP) effects. The present figure shows the mean connectivity patterns (averaged across the subjects) for the target RS networks. (**a**) in the upper row represents the connectivity pattern, in the alpha band, of the response-implementation network predicting, using a support vector regression (SVR), the behavioral performance (delta global reaction time (RT) and inverse efficiency score (IES)). (**b**) represents the resting network activity of the expectancy-implementation network in the theta band, predicting delta global accuracy. The lower row represents the RS-FC predicting the task-dependent ERP modulations. Specifically, functional connectivity at rest in the response-implementation network predicted the response-implementation ERP effects (P3 amplitude increase) in the theta frequency band (**c**). Similarly, the RS-FC of the expectancy-violation network in the delta band predicted the ERP effect (omission detection potential (ODP) increase) elicited during the expectancy violation in the task (**d**). To avoid overloading of images, we report only the main results here. Additional figures are provided in the Supplementary Materials.

**Figure 6:**
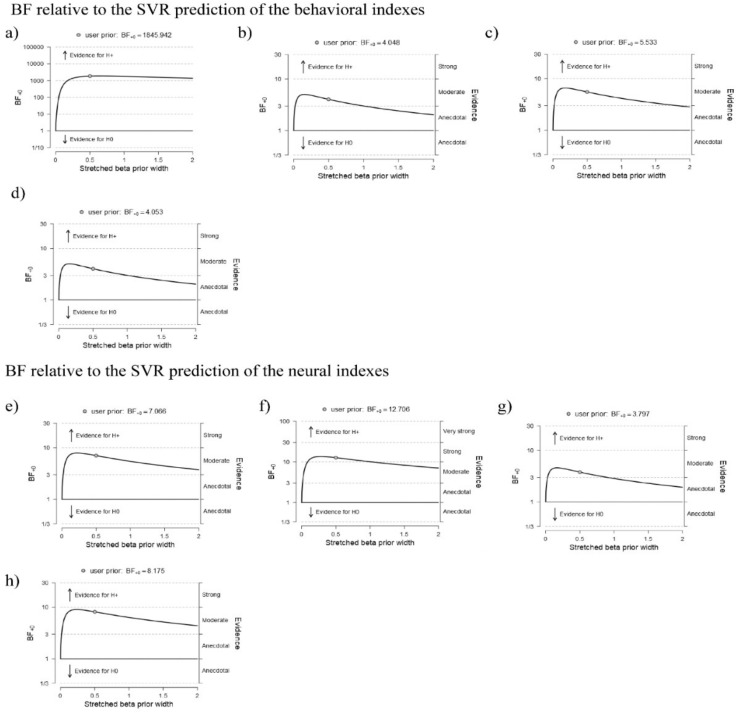
Robustness check of Bayesian correlation in SVR prediction. The present figure shows the robustness check graphs of the Bayes Factor reported in the main text. Each panel shows a robustness check for the respective correlation reported in manuscript. The graph highlights the variation of the Bayes Factor (BF) in dependence of the beta prior width providing a check of the reliability of the result. The evidence is classified based on the BF magnitude. Here we report the BF10 showing the probability of the alternative hypothesis (1) vs. the probability of the null hypothesis (0). Namely, the higher the BF, the higher the probability of the alternative hypothesis. (**a**) robustness check of the connectivity in the response implementation network predicting the behavioral delta global accuracy in the theta band, and the behavioral delta global RT and the delta global inverse efficiency score (IES) in the alpha band respectively in (**b**,**c**). In (**d**) the robustness check of the BF of the connectivity patterns within the expectancy-implementation network in the theta band predicting the delta global accuracy. In (**e**) the robustness check of the BF of the connectivity within the response implementation network predicting the P3 amplitude modulation (response implementation ERP effect) in the theta band. (**f**) robustness check of expectancy violation network predicting the omission-detection potential (ODP) (the expectancy-violation ERP effect) in the alpha, beta (**g**) and delta (**h**) frequency bands.

The SVR results showed that all the behavioral indexes could be predicted from the connectivity pattern measured within the response implementation network. In particular, the delta global accuracy was significantly predicted only in the theta frequency band (R = 0.58; BF10 = 1845.94; c.i. = (0.31, 0.72); see Supplementary Figure S1a), whereas in the alpha band, both the delta global RT (R = 0.31; BF10 = 4.04; c.i. = (0.05, 0.52); Figure 5a; for the robustness check see Figure 6a,b) and the delta global inverse efficiency score (IES) (R = 0.33; BF10 = 5.53; c.i. = (0.06, 0.54]; see Figure 5a; for the robustness check see Figure 6c) could be predicted. Moreover, the connectivity patterns within the expectancy-implementation network in the theta frequency band predicted the delta global accuracy (R = 0.31; BF10 = 4.05; c.i. = (0.05, 0.52); see Figure 5b; for the robustness check see Figure 6d).

### 3.2. Predicting Neural Effects from the Networks of Interest

The application of SVR on the neural measures revealed that the RS-FC measured within the response implementation network was able to predict the P3 amplitude modulation (response implementation ERP effect) in the theta frequency band (R = 0.37; BF10 = 7.06; c.i. = (0.07, 0.58); see Figure 5c; for the robustness check see Figure 6e). We also observed that expectancy violation network RS-FC predicted the task-dependent modulation of the omission-detection potential (ODP) (the expectancy-violation ERP effect) in the alpha (R = 0.40; BF10 = 12.70; c.i. = (0.10, 0.61); see Supplementary Figure S1b; for the robustness check see Figure 6f), beta (R = 0.32; BF10 = 3.79; c.i. = (0.05, 0.55); see Supplementary Figure S1b; for the robustness check see Figure 6g) and delta (R = 0.38; BF10 = 8.17; c.i. = (0.08, 0.59); see Figure 5d; for the robustness check see Figure 6h) frequency bands.

### 3.3. Correlations between the Local Graph Indexes and Behavioral Effects

**Figure 7:**
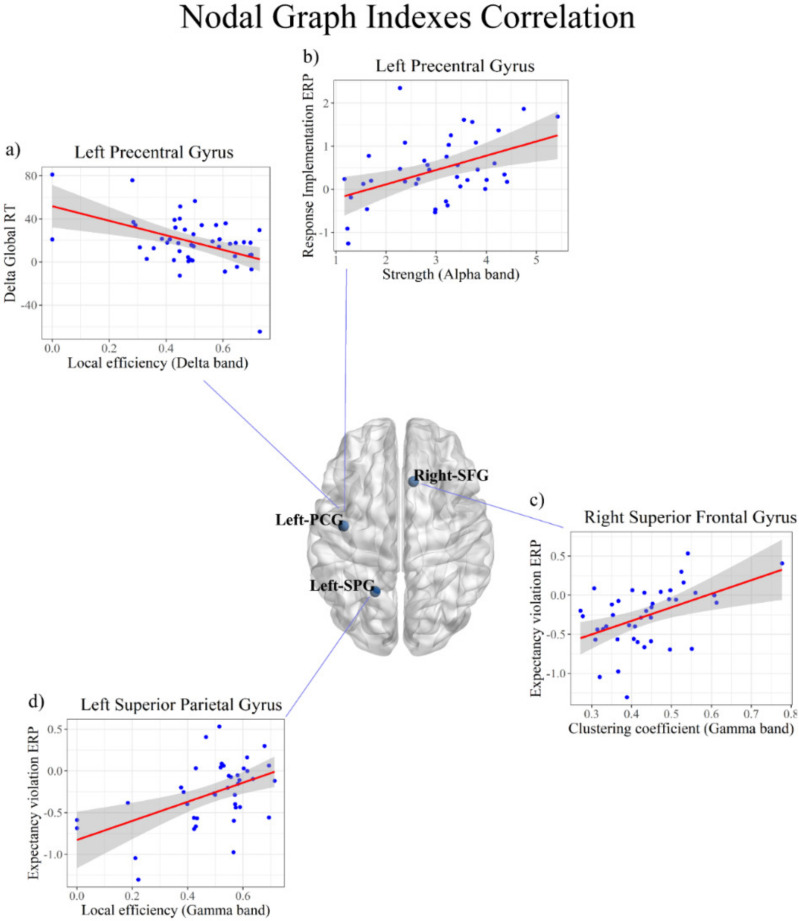
Correlation between the nodal graph indexes and task-dependent behavioral and ERP effects. The pictures represent the significant correlations between the nodal graph indexes and behavioral or ERP effects. The shaded area around the regression line represents the 95% confidence interval. (**a**) correlation of the local efficiency in the delta band of the left Precentral Gyrus (left-PCG) with the behavioral performance (delta global RT). In (**b**) it is showed the correlation of the left-PCG nodal strength in the alpha band and the amplitude of the task-dependent P3 modulation (expectancy implementation). (**c**) correlation between clustering coefficient of the right Superior Frontal Gyrus in the gamma band and the omission detection potential (ODP) increase (expectancy violation). (**d**) correlation in the gamma band between left Superior Parietal Gyrus and task-dependent expectancy violation potential.

**Figure 8:**
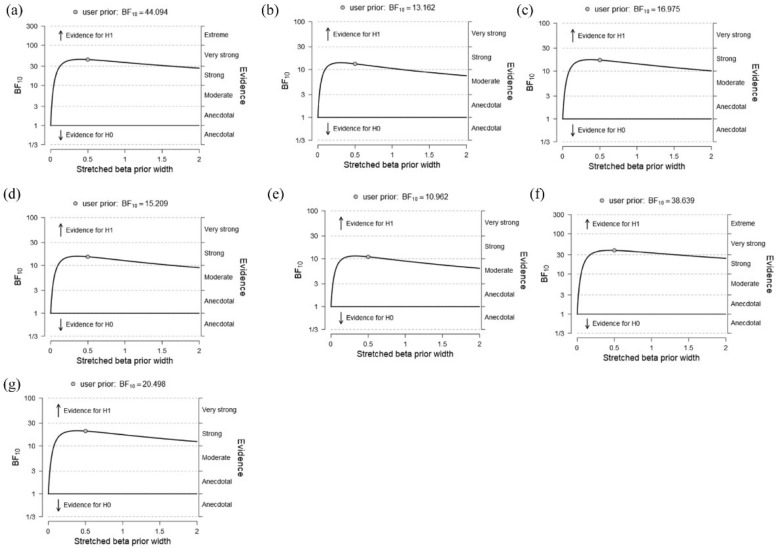
Robustness check of Bayesian correlations between the graph theory indexes and the behavioral and neural task-related measures. The present figure shows the robustness check graphs of the Bayes Factor reported in the main text. Each panel shows a robustness check for the respective correlation reported in manuscript. The graph highlights the variation of the BF in dependence of the beta prior width providing a check of the reliability of the result. The evidence is classified based on the BF magnitude. Here, we report the BF10 showing the probability of the alternative hypothesis (1) vs. the probability of the null hypothesis (0). Namely, the higher the BF, the higher the probability of the alternative hypothesis is. (**a**) BF of the correlation delta global Reaction Time and the left Precentral Gyrus (left-PCG) local efficiency; (**b**) BF of the correlation between delta global inverse efficiency score (IES) and the left-PCG local efficiency; (**c**) BF of the correlation between response implementation potential (ERP) effect (P3 modulation) and the left-PCG strength; (**d**) BF of the correlation between response implementation ERP effect (P3 modulation) and the left-PCG degree; (**e**) BF of the correlation between expectancy violation ERP effect (omission detection potential (ODP) modulation) and the right-PCG local efficiency; (**f**) BF of the correlation between expectancy violation ERP effect (ODP modulation) and the left Superior Parietal Gyrus local efficiency; (**g**) BF of the correlation between expectancy violation ERP effect (ODP modulation) and the right-PCG clustering coefficient.

The results in the delta band showed a strong negative correlation (BF10 = 44.09) between the local efficiency in the left precentral gyrus (left-PCG) and both the delta global RT (R = −0.47; c.i. = (−0.64, −0.19); see Figure 7a) and delta global IES (BF10 = 13.16; R = −0.42, c.i. = (−0.61, −0.13); see Supplementary Figure S2a). The robustness check showed that the evidence for the alternative hypothesis (H1) remained strong when varying the beta prior width, supporting the robustness of our results (see Figure 8a,b).

### 3.4. Correlations between the Local Graph Indexes and Neural Effects

The correlational analysis between the local graph indexes and the neural effects showed strong positive correlations in the alpha and gamma frequency bands. In particular, in the alpha frequency band, we found a strong positive correlation (BF10 = 16.97) between the strength computed in the left-PCG and the response implementation ERP effect (P3 modulation) observed in the task (R = 0.46; c.i. = (0.15, 0.65); see Figure 7b). A similar positive correlation emerged between the degree computed in the same node and the task-dependent P3 modulation (BF10 = 15.21; R = 0.45, c.i. = (0.15, 0.64); see Supplementary Figure S1b). The robustness checks (see Figure 8c,d) showed that the evidence for the alternative hypothesis (H1) remained strong when varying the beta prior with, supporting the robustness of our results. Furthermore, in the gamma frequency band, we found a strong positive correlation between the local efficiency computed in the right superior frontal gyrus (right-SFG) and the ERP effect of the expectancy violation (ODP modulation) induced by the task (BF10 = 10.96; R = 0.44, c.i. = (0.12, 0.63); see Figure 7c) as well as between the clustering coefficient and the ODP modulation (BF10 = 20.5; R = 0.47, c.i. = (0.16, 0.65); Supplementary Figure S2c).Finally, a positive correlation emerged between the local efficiency computed in the left superior parietal gyrus (left-SPG) and the ODP modulation induced by the task (BF10 = 38.64; R = 0.50, c.i. = (0.19, 0.67); see Figure 7d). The robustness checks (see Figure 8e,f,g) showed that the evidence toward the alternative hypothesis (H1) remained strong when varying the beta prior width, supporting the robustness of our results.

## 4. Discussion

Traditionally, cognitive control (or executive function) has been conceptualized as a supra-ordinate entity exerting voluntary top-down influences on human behavior [3]. In line with this old-fashioned idea, cognitive control is deemed as a by-product of a central volitional system in charge of supervising low-level subsystems (e.g., language, perception, numerical and social cognition, movement, etc.) in order to deliberately achieve a goal, overriding automatic tendencies to behave or think according to our habits. However, a new theoretical perspective is supporting a more ecological approach positing cognitive control as grounded in associative learning. In this view, cognitive control (especially flexibility) is more properly deemed as an emerging property sensitive to environmental stimuli that can operate in the absence of awareness or top-down attention [12]. In this regard, studies suggested that “low-level” learning and conditioning mechanisms may influence cognitive control [64]. Hence, stimuli in the environment can trigger cognitive control bottom-up [65], allowing for a more efficient allocation of control strategies. The ability to “capitalize” bottom-up resources to generate adaptive cognitive control is therefore essential for adjusting behavior as a function of both internal instances and external requests, avoiding being at the mercy of environmental events. In line with this, our experimental task specifically allows us to highlight not only the presence of associative learning but the influence that the latter exerts on proactive motor control. In fact, our participants not only succeed in adapting their response speed according to the task speed but also to control behavior to avoid false alarm responses. This means that the global predictive context (block-type) is able to modulate not only excitatory mechanisms (e.g., I become faster if the task requires it) but also inhibitory ones (I control the allocation of anticipatory resources so that I become faster BUT without losing accuracy). This means that in order to perform well, one must not only demonstrate the ability to speed-up or slow-down response speed according to task speed but also to (1) update continuously the internal representation of contextual factors (i.e., task speed) and (2) regulate accordingly behavior in a proactive way. Importantly, unlike other experimental designs traditionally used to test associative learning (i.e., artificial grammar language or serial reaction times), the DTP task introduces the possibility of measuring the degree of flexibility with which a given piece of implicitly learned information is updated bottom-up according to contextual aspects. In other words, the task we used is not limited to measuring implicit learning but specifically taps on the degree of flexibility of the latter. This aspect represents a novel element in the literature [13,14] for further discussion.

In the present study, we step further and address whether adaptive cognitive control is grounded in the brain’s intrinsic organization. We used the source-resolved oscillatory HD-EEG activity recorded at rest to measure the intrinsic FC. We then used this measure to predict the behavioral and neural effects elicited by a task requiring implicit, flexible motor control (i.e., the DTP task). Remarkably, this approach allowed different frequency bands to be targeted, deepening the functional link between rest and task oscillatory activity. We drew upon our previous findings and targeted three specific, task-informed neural networks reflecting expectancy implementation, expectancy violation and response implementation as three distinct computational stages underlying implicit motor control [13]. We used an SVR approach to address whether the RS-FC oscillatory patterns within each of these three networks could predict both task response speed/accuracy and ERP modulations induced by global stimulus predictability. At first, we found that the RS connectivity in the theta band, measured in both the expectancy- and response-implementation networks, predicted the delta global accuracy. This index has been interpreted as a measure of one’s capability to inhibit motor schemas built upon prior experience and efficiently adapt one’s behavior to new environmental requests in order to prevent errors (i.e., premature responses) [14]. This finding is in line with the alleged core role of the theta band for cognitive control: it may be assumed to convey the feedback information that something needs to be done when an unexpected, upcoming event occurs that violates a previously established predictive model [13,65,66,67]. In the DTP context, which implies implicit and flexible motor control, this may suggest that theta band RS-FC can predict the system’s future capability to detect the covert changes in the task speed and adapt to them flexibly. This will turn into updating a motor schema implicitly built up from experience (e.g., preparing for a probable early response in fast blocks) into a new one (e.g., preparing for a probable late response in slow blocks) in order to optimize one’s behavior. Our findings suggest that these mechanisms can be at least partially accounted for by the theta oscillatory status at rest of two distinct cortical circuits, namely the expectancy- and the response-implementation networks. Interestingly, both of these networks include frontal and parietal areas that previously have been associated with either top-down or bottom-up orienting of attentional and motor resources over time [27,28,68,69]. Moreover, we found that alpha band RS-FC in the response implementation network predicted the response speed task-adaptation either when this was considered alone (delta global RT) or corrected for the speed-accuracy trade off (delta global IES). Alpha frequency, a predominant band in the RS EEG, recently has been associated with the activity of the BOLD-derived RS networks [70,71,72]. Furthermore, this band has also been related to network efficiency during the task in at least two ways. On one hand, trial-by-trial alpha prestimulus fluctuations predict performance efficiency in cognitive tasks [73,74]. On the other hand, the RS alpha power of a given network is inversely correlated with its own task-related cerebral blood flow [75]. This pattern may suggest an efficient allocation of metabolic resources to process incoming stimuli due to higher preactivation (RS alpha power) of the task-relevant networks. Our results fit nicely with this framework, suggesting that the alpha pre-activation of the response-implementation network predisposes the system toward better computational efficiency, thus optimizing motor behavior (i.e., faster RTs in the fast blocks). Taken together, these findings suggest that the source-resolved HD-EEG can be a useful tool for investigating the role of intrinsic brain organization (RS-FC) in predicting the behavioral correlates of cognitive functioning and, in particular, of implicit motor-control mechanisms.

In addition, we also explored the relationship between RS-FC and task-evoked neural activity. Specifically, the SVR highlighted significant predictions of the theta-band RS-FC regarding the task-driven ERP modulations. Indeed, the intrinsic oscillatory organization of the response implementation network at rest partially accounted for the larger P3 modulation observed in fast vs. slow blocks. This finding converges with behavioral data and confirms that RS theta activity potential explains at least part of the variability in implicit motor-control adaptation. Moreover, we found an additional link between the RS-FC calculated within the expectancy violation network and its task-related activation (i.e., modulation of the omission detection potential). Overall, these findings suggest that the RS-FC of a given network is predictive of its subsequent task-related activity, in line with previous neuroimaging studies [70,76,77,78]. Notably, the present results align nicely with our previous ones [13], which showed task-related theta and delta synchronization for response implementation and expectancy violation, respectively. This frequency mirroring between resting and task-network activity suggests a possible link between the inner functional communication structure among network nodes and the system’s oscillatory perturbation during the task. Besides looking at the global connectivity patterns, we also tried to unravel the specific contributions of all single nodes common to the three considered networks (i.e., the intersection network) by using graph-derived local measures. This analysis revealed a negative Bayesian correlation in the delta band between the local efficiency of the left-PCG and behavioral measures (both the delta global RT and IES indexes). Local efficiency indicates how effectively information is integrated among the immediate neighbors of a given network node [57]. We observed that the local efficiency increased when the two behavioral indexes decreased. Notably, lower behavioral delta scores indicate that the participant has efficiently adapted his or her performance to the implicit task demand [14]. Hence, this finding suggests that the intrinsic information exchange and elaboration efficiency of the left motor area (i.e., that is contralateral to most of the participants’ handedness) are related to an individual’s ability to flexibly adjust his or her proactive motor control. Previous studies proposed that the delta band may play a crucial role for large-scale cortical integration [79,80]. In light of this, the increase in the delta band’s local efficiency may represent a possible functional mechanism for integrating the information afferent from other nodes into the dominant motor area to regulate the motor program proactively [81,82]. In other words, the more efficiently the left motor area is intrinsically interconnected, the more successful the system will be in flexibly implementing implicit motor control. In support of this hypothesis, we also report that other local measures of this node (i.e., the strength and degree) were significantly correlated with the ERP modulation related to response implementation (P3). The graph analysis further disclosed a positive correlation between the nodal measures (local efficiency and clustering coefficient) in the gamma band of two regions of the intersection network, namely the right-SFG and the left-SPG, and the ERP signature of expectancy violation (ODP). The gamma band is generally involved in attention, memory and cognitive control [83,84], but it is also considered to mediate information integration [85,86]. In this view, a possible explanation of our findings is that these two nodes operate as network hubs (with high clustering coefficients and local efficiency) in the gamma band to optimize resource allocation when any potential bottom-up conflict between the internal prediction and the external inputs arises [87,88]. In the DTP task, this occurs when an implicit temporal expectation is violated by a delayed stimulus onset (reflected by the ODP amplitude).

## 5. Conclusions

In conclusion, in this study, we propose a methodological approach based on the application of source-resolved functional connectivity to investigate the relationship between the intrinsic functional brain organization (RS-FC) and both behavioral and neural correlates elicited in the context of a motor task requiring implicit flexibility. These findings also have theoretical implications for understanding the nature of cognitive control because they follow the evidence that similar neural correlates are engaged upon explicit and implicit task demand [13]. Indeed, our findings indicate that a partial overlap between top-down and bottom-up control functional mechanisms is already grounded in the brain’s intrinsic oscillatory organization. In the present study, we focused on specific cortical networks, which were identified in our previous work [13] in relation to the three computational stages involved in the DTP task namely, expectancy implementation, expectancy violation and response implementation. We previously reported possible overlapping between the cortical areas engaged in these processes and the neuroimaging literature of cognitive control and temporal expectancy. Additional studies are needed to test the possibility to generalize our results to other experimental tasks. Moreover, future studies could expand upon the present findings to fit not only group level performance but trying to implement personalized models in order to predict individual performance based on the intrinsic brain connectivity.

## Data Availability

Resting state EEG data, connectivity matrices, task related behavioral and ERP measures and analysis code are freely accessible on Open Science Framework (https://osf.io/xw8nq/, accessed on 14 November 2021).

## Supplementary Materials

The following are available online at https://www.mdpi.com/article/10.3390/brainsci11111513/s1, Figure S1: RS-FC predicting task-dependent behavioral and ERP effects. Figure S2, Correlation between the nodal graph indexes and task-dependent behavioral and ERP effects. Table S1, In the present table are listed the Destrieux Atlas nodes, as named in the Brainstorm software, forming each of our target networks.
