# Supplementary material for: Grounding Adaptive Cognitive Control in the Intrinsic, Functional Brain Organization: An HD-EEG Resting State Investigation
Source: Brain Sci. 2021 Nov 15;11(11):1513. doi: 10.3390/brainsci11111513 (PMC8615880; doi:10.3390/brainsci11111513)
Supplement: Supplementary file 1 [file brainsci-11-01513-s001.zip › brainsci-1431770-supplementary.pdf]

Supplementary material of: **Grounding adaptive cognitive control in the intrinsic, functional brain organization: an HD-EEG resting state investigation**

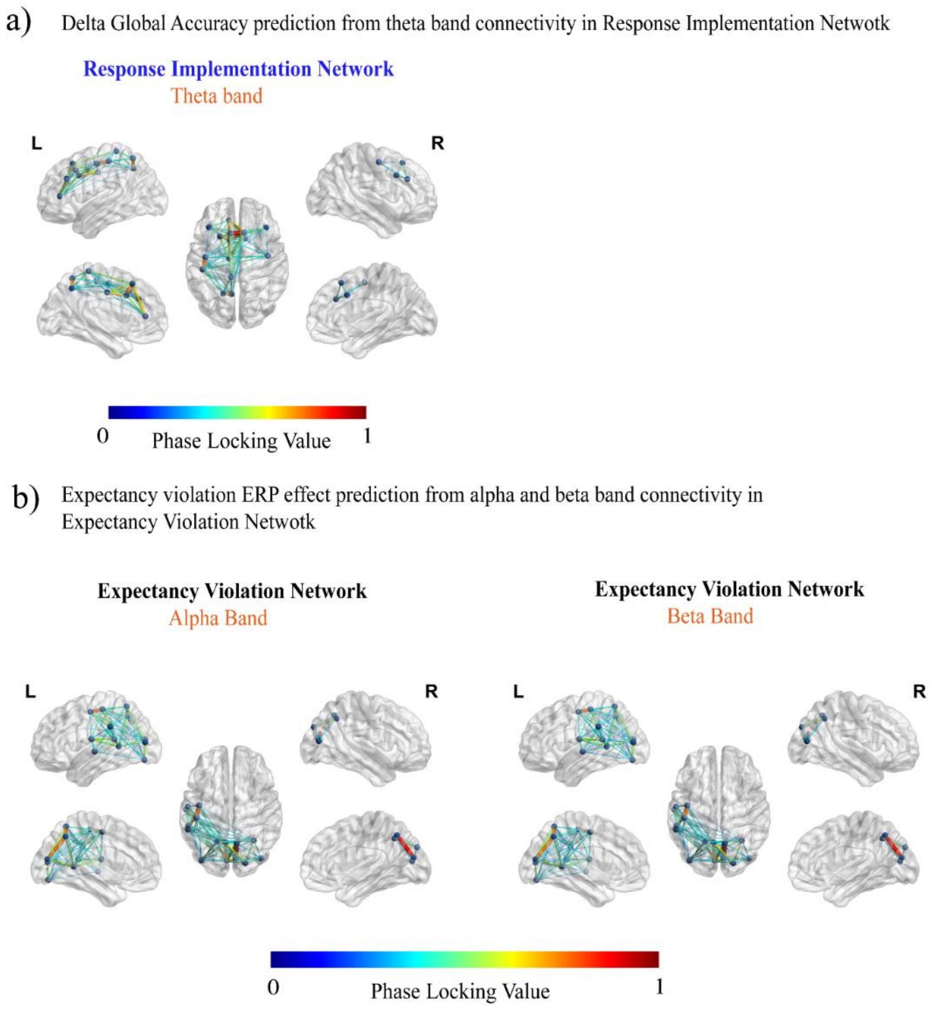

Supplementary Figure S1. RS-FC predicting task-dependent behavioral and ERP effects. The present figure shows the mean connectivity patterns (averaged across the subjects) for the target RS networks. Panel a) in the upper row represents the connectivity pattern, in the theta band, of the response-implementation network predicting behavioral performance (delta global Accuracy). Panels b and c represent the resting network activity of the expectancy-violation network in the alpha and beta band predicting the task dependent expectancy-violation ERP effect (ODP).

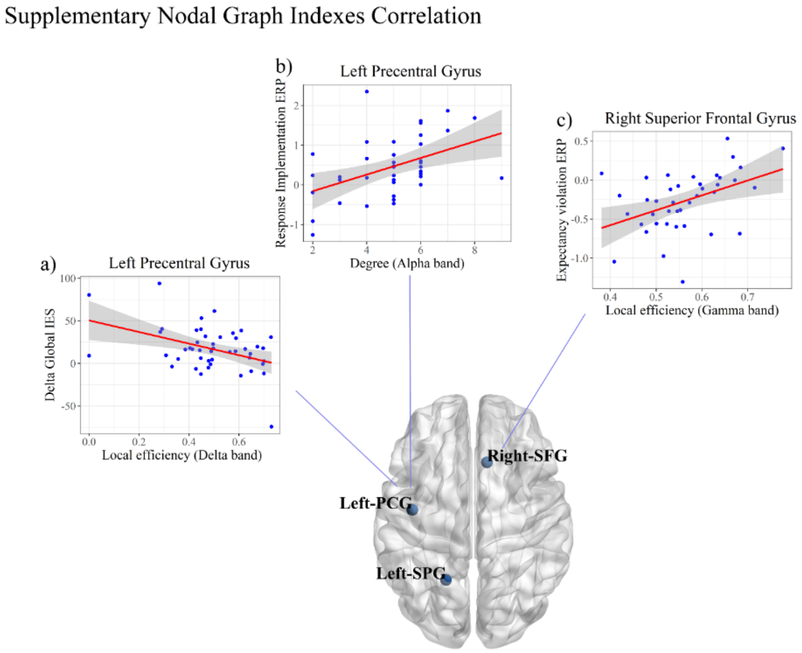

Supplementary Figure S2. Correlation between the nodal graph indexes and task-dependent behavioral and ERP effects. The pictures represent the significant correlations between the nodal graph indexes and behavioral or ERP effects. The shaded area around the regression line represents the 95% confidence interval.

Expectancy Implementation network (32 ROIs)	Expectancy Violation network (17 ROIs)	Response Implementation network (15 ROIs)
G_and_S_cingul-Mid-Ant L<br>G_and_S_cingul-Mid-Ant R<br>G_and_S_cingul-Mid-Post L<br>G_and_S_cingul-Mid-Post R<br>G_and_S_frontomargin R<br>G_and_S_occipital_inf L<br>G_and_S_paracentral L<br>G_cingul-Post-dorsal L<br>G_cingul-Post-dorsal R<br>G_cuneus L<br>G_cuneus R<br>G_front_middle L<br>G_front_middle R<br>G_front_sup L<br>G_front_sup R<br>G_oc-temp_med-Lingual L<br>G_oc-temp_med-Lingual R<br>G_oc-temp_med-Parahip R<br>G_occipital_middle L<br>G_occipital_sup L<br>G_occipital_sup R<br>G_pariet_inf-Angular L<br>G_pariet_inf-Supramar L<br>G_parietal_sup L<br>G_postcentral R<br>G_precentral L<br>Pole_occipital L<br>Pole_occipital R<br>S_front_sup R<br>S_intrapariet_and_P_trans L<br>S_intrapariet_and_P_trans R<br>S_oc_middle_and_Lunatus L	G_and_S_occipital_inf L<br>G_and_S_subcentral L<br>G_cuneus L<br>G_cuneus R<br>G_occipital_middle L<br>G_occipital_middle R<br>G_occipital_sup R<br>G_pariet_inf-Supramar L<br>G_parietal_sup L<br>G_postcentral L<br>G_precentral L<br>G_precuneus L<br>G_precuneus R<br>G_temp_sup-Lateral L<br>G_temp_sup-Plan_tempo L<br>S_intrapariet_and_P_trans R<br>S_temporal_sup L	G_and_S_cingul-Ant L<br>G_and_S_cingul-Mid-Ant L<br>G_and_S_cingul-Mid-Ant R<br>G_and_S_cingul-Mid-Post L<br>G_and_S_paracentral L<br>G_front_middle L<br>G_front_middle R<br>G_front_sup L<br>G_front_sup R<br>G_parietal_sup L<br>G_postcentral L<br>G_precentral L<br>G_precentral R<br>G_precuneus L<br>S_front_sup R

Supplementary Table S1. In the present table are listed the Destrieux Atlas nodes, as named in the Brainstorm software, forming each of our target networks.
